# Physical Therapy in Wound Care: A Cost-Effectiveness Analysis

**DOI:** 10.1097/MD.0000000000002202

**Published:** 2015-12-11

**Authors:** Kehua Zhou, Kenneth Krug, Michael S. Brogan

**Affiliations:** From the Daemen College Physical Therapy Wound Care Clinic, Daemen College, Amherst, New York, USA (KK, KZ); Department of Health Care Studies, Daemen College, Amherst, New York, USA (KZ); and Department of Physical Therapy, Daemen College, Amherst, New York, USA (MSB).

## Abstract

Management of chronic wounds remains unsatisfactory in terms of treatment cost and time required for complete wound closure (CWC).

This study aimed to calculate the healing rates, estimated cost, and time required for CWC in wounds; compare estimated wound care costs between healing and nonhealing wounds; and compare cost effectiveness between venous leg ulcer (VLU) and non-VLU.

This was a retrospective cohort study performed at a physical therapy (PT) wound care clinic. Deidentified patient data in the electronic medical database from September 10, 2012 to January 23, 2015 were extracted.

Among 159 included patients with wounds, 119 (74.84%) patients were healed with CWC. The included patients were treated for 109.70 ± 95.70 days, 29.71 ± 25.66 visits, and at the costs per treatment episode of $1629.65 ± 1378.82 per reimbursement rate and $2711.42 ± 2356.81 per breakeven rate. For patients with CWC (healing group), the treatment duration was 98.01 ± 76.12 days with the time for CWC as 72.45 ± 64.21 days; the cost per treatment episode was $1327.24 ± 1143.53 for reimbursement rate and $2492.58 ± 2106.88 for breakeven cost. For patients with nonhealing wounds, treatment duration was found to be longer with costs significantly higher (*P* < 0.01 for all). In the healing group, no differences were found between VLU and non-VLU in treatment duration (95.46 days vs. 100.88 days, P = 0.698), time for CWC (68.06 days vs. 77.38 days, *P* = 0.431), and cost ($2756.78 vs. 2397.84 for breakeven rate, *P* = 0.640) with the exception of wound dressing costs ($329.19 vs. 146.47, *P* = 0.001).

Healing rates may be affected with patient exclusions. Costs at physicians’ offices were not included.

Incorporation of PT in wound care appeared to be cost effective. PT may thus be a good referral option for patients with wounds. However, the results should be interpreted cautiously and further studies are warranted.

## INTRODUCTION

Open wounds can be either acute or chronic. Acute wounds usually heal in a timely manner; whereas chronic wounds have disrupted healing processes from aging, pathophysiologic, or metabolic factors.^1^ While acute wounds are often the result of traumatic or surgical events, chronic wounds are commonly caused by compromised venous circulation, jeopardized arterial supply, and/or continuous pressure. The status of wounds can impair an individual's mobility, activities of daily living, and quality of life. These issues become more significant in patients with chronic wounds and may lead to a number of complications including disability or need for assisted living, home care, depression, loss of digit or limb, infection, or even death.^2^ Approximately 60 million people worldwide are being treated for chronic and nonhealing wounds.^3^ The public health and economic impact of chronic wound care including lost work time and impaired quality of life is staggering, with an estimated annual cost upwards of 8 billion (US) dollars.^3,4^

Based on etiologies, wounds are usually classified as venous leg ulcer (VLU), arterial, diabetic foot ulcer (DFU), pressure ulcer, traumatic, surgical, burn, autoimmune, and others less common. Management of chronic wounds remains unsatisfactory in terms of treatment cost and time required for wound closure. Venous insufficiency and venous wounds account for the majority (70–90%) of lower extremity ulcers.^5^ With compression therapy, 35% to 50% of VLU remain unhealed after 6 months of treatment.^6^ Average cost of VLU is approximately $4000 per month per patient and $16,000 per treatment episode with an additional cost of up to $29,252 for some advanced wound dressings.^7^

Other wounds are not as common as VLU, but the cost of care is also astounding. For direct treatment costs of DFU in 2012, the mean cost per patient per treatment episode was estimated to range from $9650 to $19,431 and is increasing on a yearly basis.^8^ With conventional therapy, 91.7% of patients with DFU have been reported to heal without amputation and the healing rates at 12, 20, and 52 weeks were reported to be 59.3%, 70.5%, and 86.6%, respectively.^9^ The cost of pressure ulcer is high and healing rate remains low. Research in 1999 found that each Stage III or Stage IV pressure ulcers costs $14,000 to $23,000 and these numbers are expected to be higher now as the cost of care increases^10^; the complete wound closure (CWC) rate for pressure ulcer remains low with only 17% in 112 days of treatment as reported in 2011.^11^

These previous studies provide a basic understanding regarding wound healing costs and rates in various types of wounds treated under conventional physician settings or integrative physician–nursing settings.^6–11^ However, little information on wound healing costs, healing rates for CWC, and wound healing trajectories is available in multidisciplinary settings when physical therapy (PT) is involved. Wound care by physical therapists may hold advantages because of the clinicians’ familiarity and expertise with tissue repair, the use and effect of various physical agents, including electrical stimulation. Electrical stimulation has been widely researched and proven to be effective for enhancing closure in wounds by facilitating numerous biochemical, vascular, and cellular events; subsequently, electrical stimulation is widely used in PT wound care settings.^12–14^ Thus, participation of physical therapists in wound care may provide increased benefit to the patient.

Treatment costs of wound care at a PT wound clinic often depend on the aggregate use of electrical stimulation, other physical agents, dressings, debridement, and patient education. Others like therapeutic exercise and gait training may be applied but are not usually billed under wound care. Reimbursement for these services and various interventions are relatively inexpensive, which may prove PT a good referral option for the management of chronic wounds. Although randomized controlled trials have been carried out and one meta-analysis has reported better outcome in wound healing using electrical stimulation,^14^ little is known regarding the healing rates, cost, and time required for CWC in different types of wounds when PT are involved.

The purpose of this article is to present the opportunity that inclusion of PT may have to a comprehensive wound care team. This study aimed to calculate the healing rates, estimated cost, and time required for CWC in wounds; compare estimated wound care costs between healing and nonhealing wounds; and compare cost effectiveness between VLU and non-VLU (DFU, pressure ulcer, traumatic, surgical, and other types of wounds) in a PT outpatient wound care clinic.

### Participants and Methods

This was a retrospective cohort study performed at the Daemen College PT Wound Care Clinic. The study protocol was approved with an exemption by the Daemen College Institutional Review Board. Established in 2012 as a result of grants from private philanthropic organizations, this clinic has been operating as an outpatient PT clinic specializing in wound care and serving the Western New York community, by offering treatment free of charge to patients for a 2-year period. Although free care was provided to patients during the first 2 years of operation, therapists and researchers at the clinic have been tracking estimated costs of dressings, treatment procedures, and number of visits with additional patient demographics and wound related history using an electronic database since the opening of the clinic. Deidentified patient data in the electronic medical databases from the inception of the clinic on September 10, 2012 to January 23, 2015 were extracted.

Patients with wound(s) were all eligible to be included. Additionally, patients with the following conditions were excluded from the present study: patients currently being treated at the present clinic; patients lost to follow-up and had no more than 6 documented visits; patients with unstable vital signs which warranted hospitalization and advanced care due to comorbidities and thus were unable to continue outpatient care at the present clinic. The main reason for these exclusions is the difficulties in establishing the direct link between the status of these wounds and intervention at the clinic. In the present study, “healed/healing” refers to CWC and “nonhealing” refers to wound(s) remaining open upon discharge. Based on the healing results (CWC or not) upon discharge, patients were divided into the healing group and nonhealing group. Additionally, cost effectiveness analyses were also compared between VLU and non-VLU.

It is worth noting that the clinic was colocated with a physician's office (but different entities) and therapists consulted with the physician when needed during wound care. All patients with a few exceptions were referred from and were following up with this physician who is an infectious disease expert. However, due to difficulties in accessing data from physicians’ offices, costs at physicians’ offices were not included in the data analyses of the present study.

### Statistical Analysis

Patients’ demographics and wound treatment history were summarized and described. Costs, healing rates, time required for CWC, treatment durations, and number of visits were computed. Quantitative data were expressed with mean ± SD and were compared using a *t* test or Mann–Whitney *U* test between the healing and nonhealing groups and between VLU and non-VLU. Categorical data were summarized and compared with Fisher exact test. Odds ratios (OR) with 95% confidence interval (CI) were presented as measures of effect size. A 2-tailed *P* < 0.05 was considered statistically significant. All data were analyzed using SPSS 17.0 software (SPSS, Inc., Chicago, IL) for windows.

## RESULTS

**FIGURE 1:**
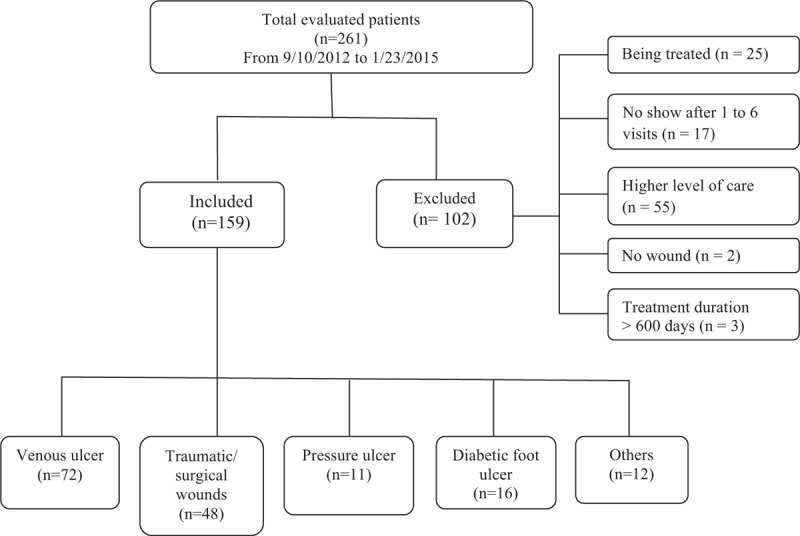
Flow chart of patient data inclusion.

From September 10, 2012 to January 23, 2015, the clinic evaluated and treated 261 patients. Among these 261 patients, 25 patients were still being treated at the clinic; 17 patients did not continue with treatment following 1 or more sessions (all were treated no more than 6 times); and 55 patients eventually were placed in higher level of care including hospitals and home care due to unstable vital signs. Additionally, 1 patient without wounds was treated at the clinic for a Morton's Neuroma; and 1 patient was treated for lymphedema without open wounds. Thus, of the 261 patients, 99 were excluded from the present study as medical documentation could not validate a direct effect or lack of direct effect between their wound status and treatment at the clinic (Fig. 1). One patient in the nonhealing group and 2 patients in the healing group were treated for an extended period beyond all others. To minimize statistic error, data on these 3 patients (n = 3) were considered outliers and were excluded from final analysis. Consequently, data on 159 patients were included for the final analyses. All patients (healing 119; nonhealing 40) were discharged per physical therapists’ judgment, physician's opinion, and/or patient's preference.

### Treatments for Wound Care

Patient data in the medical record were reviewed. All patients, with the exception of 8 patients with superficial VLU, received 45 minutes of high voltage pulsed current electric therapy (HVPC, 120 pps, 100 mA, continuous wave) as the primary treatment. Whirlpool therapy (10 minutes, 92°F patient sensory perceptive), ultrasound (nonthermal, 1 MHz, pulsed 20% duty cycle, 0.5 W/cm^2^, 3 minutes, 0.1 cm away), and ultraviolet C (UVC, 45 seconds, 0.5 cm away) therapy were occasionally used based on the clinician's evaluation of wound conditions. For example, whirlpool therapy was used when VLU was accompanied by excessive drainage and debris; ultraviolet light was used when the wound was covered with nonviable tissue; ultrasound was used for DFUs with minimal to no progress to electric stimulation and dressing change. Collagen- and silver-based dressings (donated by Medline Industries, Inc. and Derma Sciences, Inc.) were commonly applied. Additional 4-layer compression dressings together with Unna boot were used in VLU if compressible. As a clinical routine, almost all patients were treated at the clinic for 1 or more visits after CWC to prevent relapses; thus, time required for CWC differed from treatment duration, especially for patients with VLU as patients would not be discharged until they received and were comfortable with their custom-made compression garment.

### Cost Estimation

Costs in the present study included estimated reimbursement rates from insurance companies and breakeven costs for the clinic to operate plus dressing costs which was presented both as part of the breakeven cost as well as an independent item separately. As the current reimbursement rate for electric stimulation in wound care ranges from 18 to 40 dollars per patient visit, and because the occasional use of other modalities was not counted in the present study, we used a universal cost of 40 dollars per patient visit for an aggregate, estimated modality cost; and we added a $70 initial evaluation cost and $40 reevaluation (every 30 days) cost for the estimation of the reimbursed cost per treatment episode [reimbursement rate per treatment episode = ($40 × number of visits) + $70 + ($40 × number of reevaluations)]. Additionally, dressing cost, which is not usually covered by insurance companies, is estimated as the total dressing costs per treatment episode separately. As for breakeven rate, the operation of the present clinic will cost $83.00/hour, which includes salaries for 1 full-time physical therapist and 1 full-time PT aid, fringe benefits, rent, insurances, and utility bills. Thus, based on the practical schedule that 1 therapist treats 1 patient per hour (treatment of 1 patient visit typically takes around 1 hour), the breakeven cost per treatment episode = $83 × number of visits + total dressing costs.

### Demographic Characteristic and Cost Effectiveness of Wound Care

In total, 159 patients (75 males and 84 females) with an age of 63.78 ± 17.35 years were included in the present study. Among them, 119 (74.84%) patients healed (healing group) and 40 (25.16%) patients did not (nonhealing group). Seventy-one out of 159 (44.65%) patients presented with more than 1 wound at the time of initial examination, and for the purpose of analyses, only the wound with the longest duration was used for statistical comparison (Table 1).

**TABLE 1:**
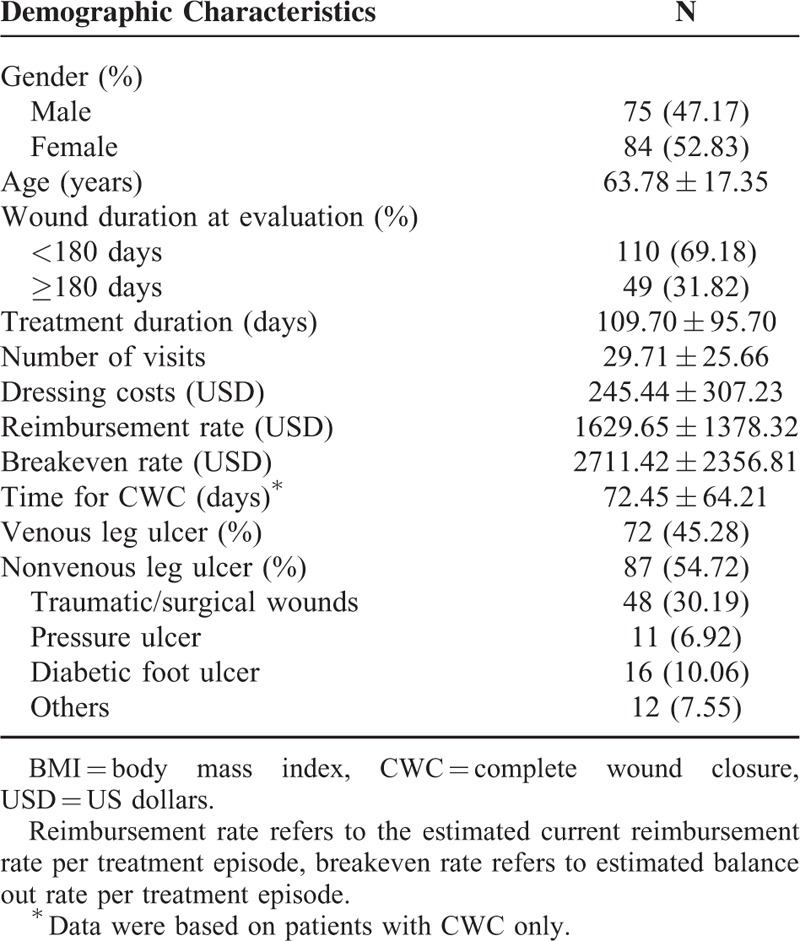
Demographic Characteristics of the Patients (n = 159)

As for wound duration at initial examination, 110 (69.18%) patients had wounds less than 180 days and 49 (30.82%) patients had wounds at least 180 days. The patients were treated for 109.70 ± 95.70 days, 29.71 ± 25.66 visits, at the costs per treatment episode of $1629.65 ± 1378.82 per reimbursement rate and $2711.42 ± 2356.81 per breakeven rate. For patients in the healed group, the time for CWC was 72.45 ± 64.21 days. Among these patients, 72 (45.28%) patients had VLU; 87 (54.72%) patients had non-VLU wounds.

### Cost Effectiveness Between Patients in the Healing and Nonhealing Groups

No significant differences were found between patients in the healing and nonhealing groups in dressing costs ($243.21 ± 301.94 vs. $252.08 ± 326.32, *P* = 0.412). However, compared to the nonhealing group, patients in the healing group had a higher proportion of VLU diagnosis (*P* = 0.001, OR = 3.88), a shorter treatment duration (98.01 ± 76.12 days vs. 144.50 ± 133.84 days, *P* < 0.001), less visit times (27.10 ± 22.64 vs. 37.48 ± 32.23, *P* = 0.001), and less cost per treatment episode in both estimated reimbursement rates ($1327.24 ± 1143.53 vs. $1751.08 ± 1536.58, *P* = 0.004) and breakeven costs ($2492.58 ± 2106.88 vs. $3362.50 ± 2914.03, *P* = 0.002) (Table 2).

**TABLE 2:**
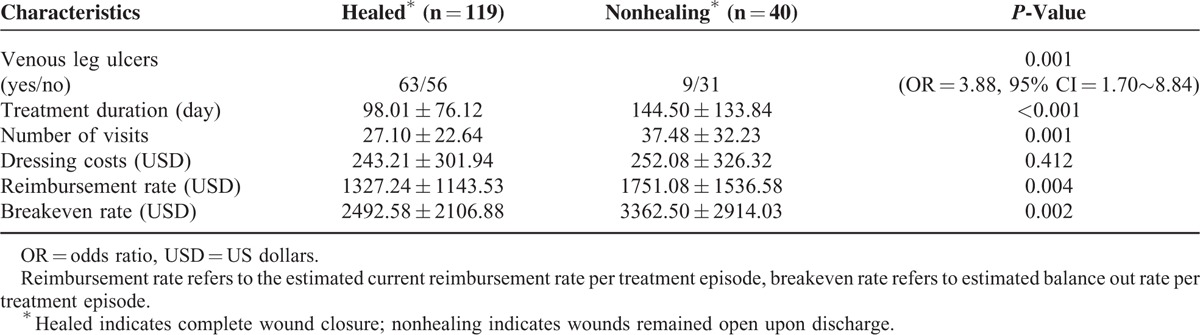
Comparison Between Healed and Nonhealing Wounds

### Cost Effectiveness Analyses Between VLU and Non-VLU Wounds

VLU is the most widely researched type of wound; thus, we compared the cost effectiveness differences between patients with VLU and patients with non-VLU wounds in the healing group. Regarding healing rates, 63/72 (87.50%) patients with VLU and 56/87 (64.37%) patients with non-VLU were in the healing group. In the healing group, patients with VLU had a wound duration of 217.76 ± 503.68 days and patients with non-VLU had a wound duration of 301.48 ± 630.46 days; no difference was found between these 2 groups (*P* = 0.429) (Table 3).

**TABLE 3:**
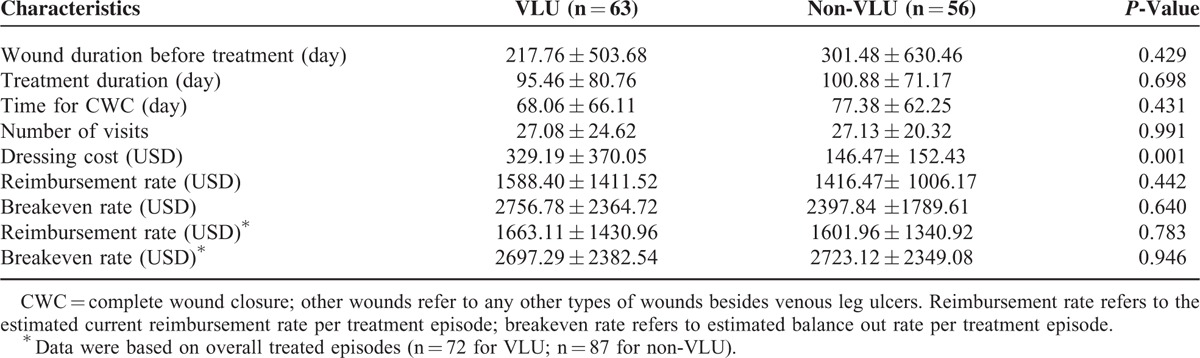
Comparison Between Venous Leg Ulcer (VLU) and Non-VLU Wounds on Patients With Healed Wounds (CWC)

Additionally, no differences were found between VLU and non-VLU in the following comparisons in the healing group: treatment duration (95.46 ± 80.76 days vs. 100.88 ± 71.17 days, *P* = 0.698), time for CWC (68.06 ± 66.01 days vs. 77.38 ± 62.25 days, *P* = 0.431), and number of visits (27.08 ± 24.62 vs. 27.13 ± 20.32, *P* = 0.991). The treatment of non-VLU took slightly longer but required almost the same number of visits (*P* = 0.698 and 0.991, respectively).

Significant differences were found between VLU and non-VLU wounds in wound dressing costs ($329.19 ± 370.05 vs. $146.47 ± 152.43, *P* = 0.001), but not for final cost per treatment episode in patients of the healing group (reimbursement rate: $1588.40 ± 1411.52 vs. $1416.47 ± 1006.17, *P* = 0.442; breakeven rate: $2756.78 ± 2364.72 vs. $2397.84 ± 1789.61, *P* = 0.640). In summary, healing of VLU and non-VLU wounds differs only in dressing costs in patients with CWC (more in VLU).

Additionally, cost of care was also calculated for all included patients in both the healing and nonhealing groups, and again, no differences were found between VLU and non-VLU wounds for reimbursement rate ($1663.11 ± 1430.96 vs. $1601.96 ± 1340.92, *P* = 0.783) and breakeven rate ($2697.29 ± 2382.54 vs. $2723.12 ± 2349.08, *P* = 0.946).

## DISCUSSION

To the best of our knowledge, this study is the first study describing healing rates and costs of wound care when PT is incorporated in the wound care team. Patients treated at the present clinic were all referred from physicians’ offices. In the present study, healing rates were 74.69% in all patients (87.50% in VLU, 64.37% in non-VLU). Time required for CWC was 72.45 ± 64.21 days (68.06 ± 66.11 days in VLU). Although it is difficult to make exact comparisons with previous research studies, such as 50% to 65% of CWC in 180 days for VLU,^6^ 20% VLU remaining unhealed at 2 years,^15^ 17% patients with CWC in 112 days for pressure ulcer,^11^ and 70.5% ulcers healed in 20 weeks for DFU,^9^ we believe the healing rates and time for CWC with the incorporation of PT are promising and inspiring for wound care.

Fife et al^16^ reported that 65.8% of wounds healed with an average time of CWC at 15 weeks (107 days; SD: 150.29). Wound care at the present study demonstrated a healing rate of 78.57% over 109.70 ± 95.70 days of treatment. The time required for CWC is shorter and the healing rate is higher in the present study as compared with the results of the previous study at a pure physician-based setting.^16^ Almost all patients treated at the present clinic received electric stimulation which is not usually used at physician based settings when PT is not included; thus, the increased healing rates of wound healing may be due to the use of electric stimulation. Previous studies support the hypothesis that electric stimulation increases healing rates of wounds.^11–14^

### Comparisons Between VLU and Non-VLU Wound Healing

Results of the present study indicate that as compared with other wound diagnoses, patients with VLU are more likely to heal (OR = 3.88, *P* = 0.001), but require higher dressing costs with similar treatment duration and cost per treatment episode. To our best knowledge, no previous studies have directly compared healing rates, costs, and time required for CWC among different types of wounds. Data relative to these interests have a wide range in previous studies regarding specific types of wounds. For example, cost per treatment episode ranges from $16,000 in VLU to $23,000 in pressure ulcers,^7,10^ and healing rates range from 50% in 3 months in VLU to 59.3% in 12 weeks in DFU.^6,9^ Comparisons between VLU and non-VLU relative to the above interests are difficult. To make the actual comparisons, large-scale studies using more sophisticated and controlled designs are warranted.

At the present study, similar to other types of wounds, VLU wounds were usually treated 2 to 3 times a week. Wound care dressing costs were more expensive for VLU than non-VLU (*P* = 0.001), however. The reason is likely due to the fact that the cost of compression dressings in VLU are more expensive and are replaced each visit as compared to other ordinary dressings. Nonetheless, VLU and non-VLU share similar cost per treatment episode in the present study, as non-VLU typically took slightly longer to heal. Before the application of compressive dressings in VLU, therapists had to wait for the results of circulatory diagnostics for the lower extremities. Additionally, following CWC, therapists had to wait for the arrival of custom-made garments before discharge for patients with VLU in the healing group. Thus, results may be favorable influenced with a more efficient referral system and garment fitting strategy.

### Cost of Wound Care With the Incorporation of PT

In the present study, the cost of each patient per treatment episode is $2711.42 ± 2356.81 ($2492.58 ± 2106.88 in healed wounds and $3362.50 ± 2914.03 in nonhealing wounds) as per breakeven rates. Due to the reason that costs at the physicians’ offices are not included in the study, comparisons for costs of wound care at the present study with those at physician only settings or integrative physician–nursing settings may not be appropriate. Nonetheless, data in the present study provide us some preliminary insights regarding costs when PT is included as an intervention. In the present study, cost per treatment episode for breakeven rate was $2756.78 ± 2364.72 in VLU and $2397.84 ± 1789.61 in non-VLU in patients with CWC. Hankin et al^7^ reported that the cost for VLU per treatment episode without advanced wound dressing was $16,000. For DFU, cost per treatment episode ranges from $9650 to 19,431 in conventional care,^8^ and $50,000 (Medicare) to $200,000 (private pay) for hyperbaric oxygen therapy which is also time consuming.^17^ For pressure ulcers, the cost per treatment episode was reported to be from $14,000 to 23,000.^10^ These are reported cost of wound care at physician or integrative physician-nursing based settings. Thus, incorporation of PT in wound care may be cost saving to reach similar effectiveness.

As indicated by previous reports regarding costs of wound care,^6–11,15–17^ PT may be a cost effective referral option for physicians given the inspiring healing rates. Nonetheless, we have to recognize that the breakeven cost in the present clinic is higher than the reimbursement rate. With the current insurance company reimbursement rate for PT, the present clinic will not be able to support itself and continue operating. Similar to care provided to a patient in outpatient orthopedic PT settings, PT wound care is subject to the same limitations such as visits per year, copays, and time limitations as per reimbursement policies. Some may argue that physical therapists may increase the number of patients per hour or decrease the patient visit frequency to balance the cost; we believe that these measures may be unjustifiable for quality of care concerns and even if plausible, more patient referrals are needed from physicians as direct access is not common in wound care, nor should it be, given the critical need for medical evaluation, and diagnostics.

Given the results of this study (a relative high healing rate with low cost), the incorporation of PT in wound care may not increase the cost of wound care but rather contribute to reductions in cost and the time required for CWC in wounds. The amount of cost that it may save and the economic value may thus derive from PT wound care may be monumental if improvements in the quality of life and working abilities of patients after wound closure are taken into consideration. To cut down the cost of care in the treatments of wounds, incorporation of PT in wound care may be a solution. However, to better compare the cost effectiveness between wound care with and without the involvement of PT, cost of care at physician settings will have to be considered and better study design is needed. Nonetheless, this study illustrates the possible benefit of including physical therapists in an overall wound management system.

## LIMITATIONS

This study only includes patients treated at one outpatient PT wound care clinic. Healing rates may be affected with patient exclusions. The costs at physicians’ offices are not included in analyses. Breakeven rate was based on rates in Buffalo, NY, where cost of living is relatively low. Reimbursement rate is a rough estimation and it may differ from the actual insurance company reimbursement rate. Due to difficulties in accessing data at other wound care centers, we were unable to directly compare the cost effectiveness of wound care with and without PT services. Nonetheless, the present study provides preliminary data on the cost-effectiveness of wound care when PT is included. The results thus may be of great value to patients with wounds, healthcare administrators and policy makers, as well as insurance companies.

## CONCLUSION

Incorporation of PT in wound care appeared to be cost effective. PT may thus be a good referral option for patients with wounds. However, the results should be interpreted cautiously. To better compare the cost effectiveness between wound care with and without the involvement of PT, cost of care at physician settings will have to be considered and a better study design is needed. Additionally, comparisons with outcomes of other wound care centers are needed to evaluate the cost effectiveness of PT wound care.
